# Mechanistic insights into IL-1β-mediated progression of tendinopathy

**DOI:** 10.3389/fimmu.2025.1657285

**Published:** 2025-10-31

**Authors:** Yuchi Zhang, Jiayue Wang, Fanbo Tang, Runzhi Xian, Huanhuan Zhang, Yanlin Yuan, Guiquan Chen, Guoqiang Yang

**Affiliations:** 1 School of Physical Education, Southwest Medical University, Luzhou, China; 2 Acupuncture and Rehabilitation Department, The Affiliated Traditional Chinese Medicine Hospital of Southwest Medical University, Luzhou, China

**Keywords:** IL-1β, tendinopathy, pathogenesis, therapeutic targets, proinflammatory cytokines

## Abstract

Tendinopathy, a chronic degenerative musculoskeletal disorder characterized by pain, edema, and functional impairment, exhibits increased prevalence among elderly populations and athletes. Despite extensive research efforts targeting the attenuation of this degenerative process, clinical outcomes frequently remain suboptimal. Recent evidence underscores the critical need for more precisely targeted modulation of inflammatory pathways to improve therapeutic efficacy. Notably, the proinflammatory cytokine interleukin-1β (IL-1β) has been implicated as a prominent mediator in the pathogenesis of tendinopathy. This review synthesizes current understanding of IL-1β synthesis and its downstream signaling transduction pathways, with the primary objective of elucidating the mechanisms by which IL-1β contributes to tendinopathy progression. Through this approach, we seek to reveal novel therapeutic targets and inform improved management strategies. Although IL-1β represents a promising therapeutic candidate for tendinopathy, as evidenced by numerous investigations, current understanding of its pathogenic role is limited by several factors, including the heterogeneity of experimental models, a lack of translational studies, and insufficient evidence linking IL-1β signaling to specific clinical manifestations. Consequently, further research is essential to delineate the precise mechanisms of IL-1β involvement in tendinopathy.

## 1 Introduction

Tendinopathy is a chronic degenerative disorder characterized by debilitating pain, functional impairment, and localized edema, exhibiting heightened prevalence among elderly individuals and athletes (1). Current therapeutic strategies for tendinopathy encompass conservative and surgical interventions (2, 3). Conservative management, primarily involving rest, physical therapy, and extracorporeal shockwave therapy, remains the first-line clinical recommendation owing to its non-invasiveness (4, 5). However, these approaches exhibit notable limitations: they act primarily as symptomatic interventions—alleviating pain temporarily but failing to reverse the underlying pathological changes of tendinopathy (6). Additionally, their long-term efficacy is further compromised in chronic or severe cases, where they cannot prevent disease progression to tendon rupture; in some instances, prolonged rest may even lead to tendon atrophy, exacerbating functional impairment (7). Surgical intervention is typically reserved for cases unresponsive to conservative measures, yet it carries inherent risks (e.g., infection, adhesion formation, and prolonged post-operative recovery) and does not guarantee satisfactory long-term functional outcomes, particularly in elderly or active patients (8). Given these limitations, the development of more effective, mechanism-driven therapeutic agents has long been hampered by an incomplete understanding of tendinopathy pathogenesis. Consequently, elucidating the key molecular mediators and signaling pathways underlying tendinopathy progression is essential to overcome the shortcomings of current treatments and establish evidence-based management strategies.

Advances in tendon biology have now firmly established inflammation as a core pathogenic driver of tendinopathy, rather than a secondary byproduct (9). Specifically, dysregulated activation of inflammatory signaling molecules is implicated at every stage of the disease: from the initial phase of tenocyte dysfunction and extracellular matrix (ECM) disorganization, to progressive ECM degradation, and ultimately to the increased risk of tendon rupture (10). This recognition has spurred the exploration of therapeutic strategies targeting inflammation; however, interventions designed to achieve complete inflammatory blockade have yielded inconsistent and often disappointing clinical results. A key reason for this failure is the growing consensus that controlled, transient inflammation is not merely non-harmful but functionally essential for tendinopathy repair (11). Against this backdrop, recent evidence has shifted the therapeutic paradigm: instead of broad, non-specific suppression of inflammation, precisely targeted modulation of inflammatory responses has emerged as a more scientifically sound and clinically viable approach (12). This strategy aims to selectively abrogate the pathogenic inflammatory cascades that drive tissue destruction, while preserving the reparative inflammatory processes critical for tendon healing—addressing the fundamental limitation of earlier anti-inflammatory interventions and aligning with the need for mechanism-based therapies.

Interleukin-1β (IL-1β), a prototypical proinflammatory cytokine, plays a critical role in the pathogenesis of tendinopathy (13, 14). Accumulating evidence from cellular and animal studies underscore the therapeutic promise of IL-1β inhibition in its treatment (15, 16). However, a comprehensive understanding of the mechanisms through which IL-1β contributes to tendinopathy remains incomplete, hindering the translation of these findings into clinical applications. To address this challenge, rodent models of induced tendinopathy and *in vitro* tendon culture systems have become indispensable preclinical tools for advancing therapeutic strategies. This review begins by consolidating current knowledge on fundamental characteristics of IL-1β, including its production and activation. It then examines recent advances in elucidating the pathogenetic roles and underlying mechanisms of IL-1β in tendinopathy. By integrating these insights, this review seeks to deepen the mechanistic understanding of tendinopathy and facilitate the development of targeted therapies, thereby reinforcing the rationale for IL-1β as a therapeutic target. Further investigation is nevertheless essential to fully delineate the precise actions of IL-1β in tendinopathy.

### 1.1 Search strategy

1) Search site: Articles are forming PubMed, a database of papers on biomedical science. 2) Database: MEDLINE. 3) Keywords: IL-1β, tendinopathy, tendon disorders, inflammation, mechanisms. 4) Boolean algorithm: (“IL-1β”) OR (“Tendinopathy” OR “Tendon disorders”). 5) Retrieval timeframe: the articles we mainly searched for were published between 2015 and 2025. When referring to classic literature to describe the basic mechanisms, the publication time of these studies was before 2015, but did not exceed 20% of the publication time of the cited literature.

#### 1.1.1 Inclusion and exclusion criteria

**Figure 1:**
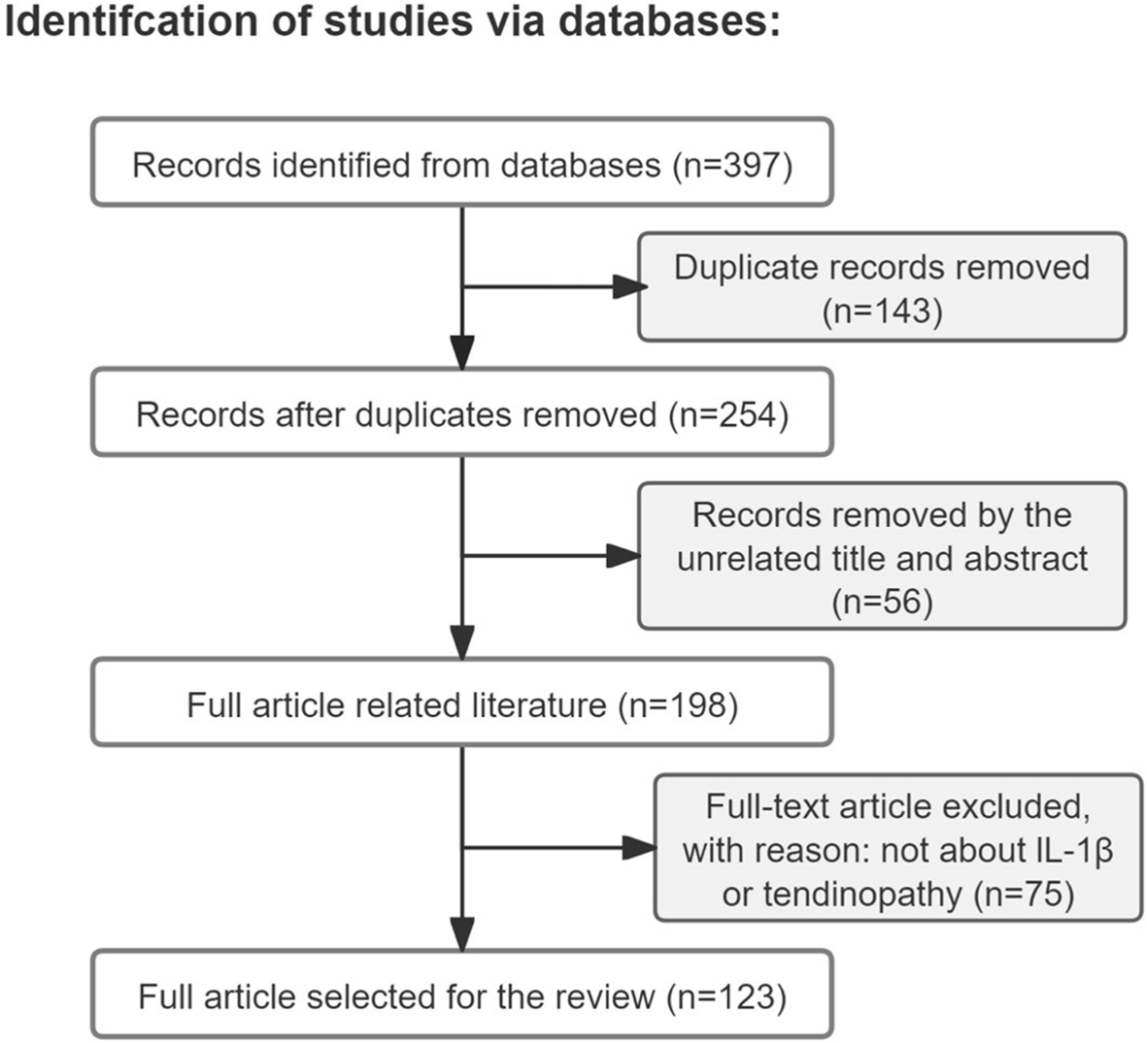
Article retrieval flow chart with inclusion and exclusion process. This flowchart illustrates the systematic procedure for identifying and selecting relevant studies. The initial database search yielded 397 records. After the removal of 143 duplicates, 254 records underwent title and abstract screening, which led to the exclusion of 56 unrelated studies. The remaining 198 full-text articles were assessed for eligibility, of which 90 were excluded for not focusing on IL-1β or tendinopathy. Ultimately, a total of 108 studies were deemed suitable and included in the review.

Articles were included if the topic is related to IL-1β or tendinopathy, and the article type was a review or experimental paper. The search process was performed as presented in **Figure 1**.

## 2 IL-1β processing and production

**Figure 2:**
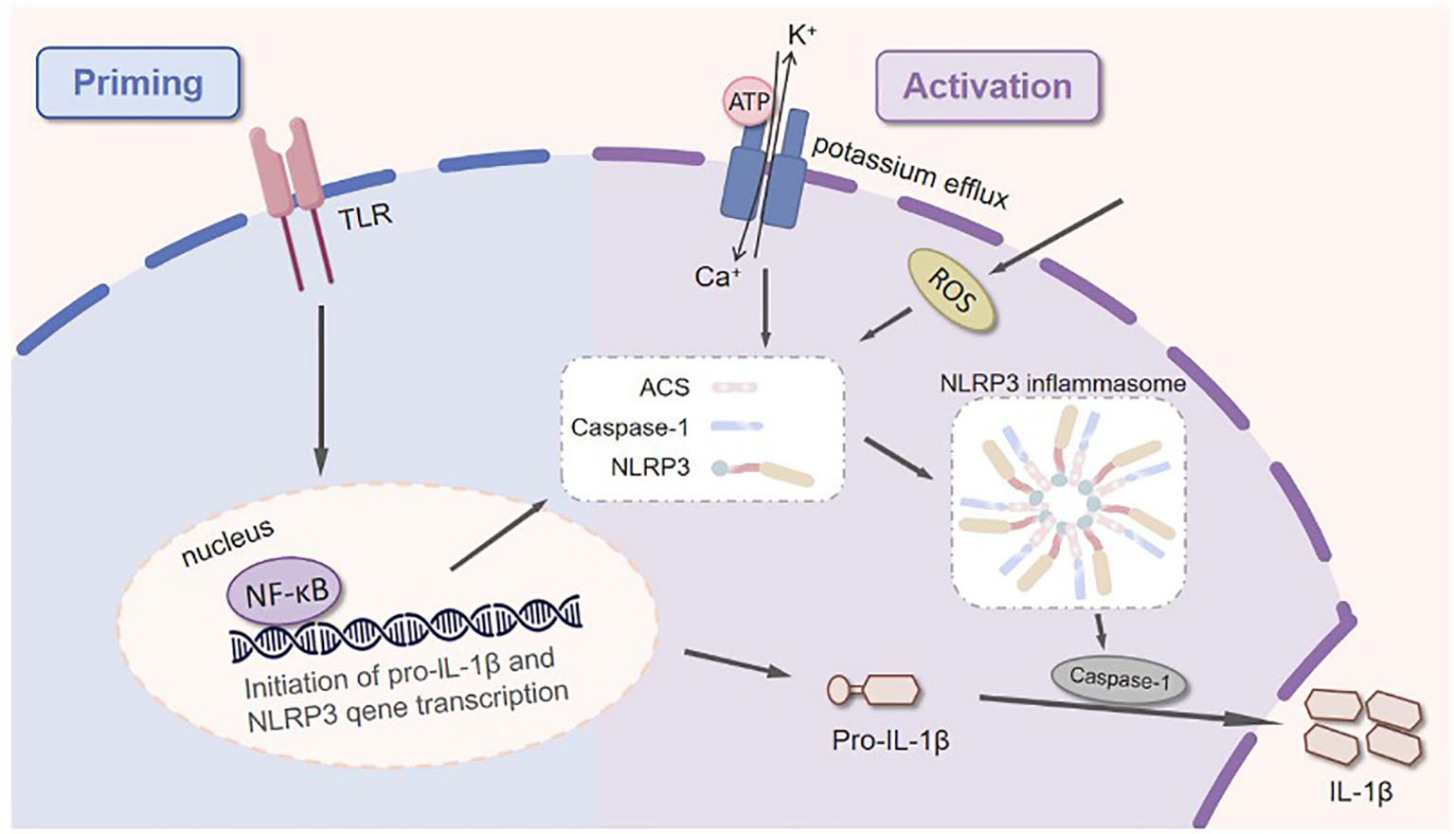
This schematic illustrates the canonical two-step mechanism underlying the maturation and release of the pro-inflammatory cytokine IL-1β. Signal 1 (Priming): Pathogen- or damage-associated molecular patterns (PAMPs/DAMPs) engage pattern-recognition receptors (e.g., Toll-like Receptors, TLRs), initiating a signaling cascade that activates the transcription factor NF-κB. This leads to the transcriptional upregulation of both pro-IL-1β and the NLRP3 protein, preparing the necessary components for inflammasome assembly. Signal 2 (Activation): Diverse stimuli, including extracellular ATP (leading to P2X7 receptor activation and K^+^ efflux) and crystalline structures, trigger the assembly of the NLRP3 inflammasome. This multi-protein complex recruits the adapter protein ASC (Apoptosis-associated speck-like protein containing a CARD), which then recruits and activates pro-caspase-1. Activated caspase-1 cleaves the inactive precursor pro-IL-1β into its biologically active form, IL-1β, which is subsequently released from the cell to drive inflammatory responses.

IL-1β is predominantly synthesized by macrophages and dendritic cells (17). However, it can also be secreted by tenocytes, tendon stem/progenitor cells (TSPCs), and osteoblasts (18). Initially, IL-1β is translated as an inactive precursor protein, pro-IL-1β, which requires proteolytic cleavage to generate the bioactive mature cytokines (19). Consequently, the production of IL-1β in most cell types follows a tightly regulated two-step process: (1) a priming step that induces the expression of IL-1β precursor and inflammasome components, and (2) an activation step that triggers NLRP3 inflammasome assembly, caspase-1 activation, and subsequent IL-1β maturation (**Figure 2**) (20). The priming cascade is initiated when pattern recognition receptors (PRRs)—most commonly Toll-like receptors (TLRs, e.g., TLR4) on the cell surface or endosomes—bind to damage-associated molecular patterns (DAMPs, such as fragmented extracellular matrix or mitochondrial DNA released by injured tendon cells) (21). Upon DAMP binding, TLRs recruit intracellular adaptor proteins (e.g., MyD88), which activate downstream signaling cascades, predominantly the NF-κB pathway. Specifically, this activation leads to phosphorylation and degradation of the NF-κB inhibitor (IκB), freeing NF-κB to translocate into the nucleus (22). Once in the nucleus, NF-κB drives the transcriptional upregulation of two key targets: (a) the inactive precursor form of IL-1β (pro-IL-1β) and (b) components of the NOD-like receptor protein 3 (NLRP3) inflammasome, including NLRP3 itself and the adaptor protein ASC (apoptosis-associated speck-like protein containing a caspase recruitment domain (23). This priming step is essential, as it ensures the cell accumulates sufficient pro-IL-1β and inflammasome components to respond to subsequent activation signals. Subsequently, a diverse range of stimuli—including reactive oxygen species (ROS) and extracellular adenosine triphosphate (ATP)—act through but complementary mechanisms to activate the NLRP3 inflammasome complex (24). Notably, ROS, generated by mitochondrial dysfunction or NADPH oxidase activation in response to tissue injury, induces oxidative stress that damages mitochondrial membranes. This damage releases mitochondrial DAMPs into the cytoplasm, which then directly interact with NLRP3, further stabilizing its oligomerization and enhancing inflammasome activation (25). On the other hand, extracellular ATP binds to the P2X7 receptor (a cation channel) on the cell membrane. This binding induces rapid opening of the P2X7 receptor, leading to massive efflux of intracellular potassium ions (K^+^). A decrease in cytosolic K^+^ concentration is a critical “danger signal” that directly promotes the oligomerization of NLRP3 proteins (a key step in inflammasome assembly) (26). Following NLRP3 oligomerization, the adaptor protein ASC is recruited via homotypic interactions (NLRP3’s pyrin domain binds ASC’s pyrin domain). ASC then acts as a scaffold to recruit pro-caspase-1, forming a large multiprotein complex known as the “NLRP3 inflammasome (27).” The assembly of this complex induces conformational changes in pro-caspase-1, driving its autocatalytic cleavage into the enzymatically active form, caspase-1 (28). Once activated, caspase-1 catalyzes the proteolytic maturation of IL-1β by cleaving its inactive precursor, pro-IL-1β, at specific aspartate residues. This cleavage generates the mature, biologically active form of IL-1β (29). Notably, since mature IL-1β lacks a conventional signal peptide, it cannot be released via the classical endoplasmic reticulum–Golgi secretory pathway. Instead, its export depends on a non-classical secretion mechanism mediated by Gasdermin D (GSDMD), which is itself a substrate of caspase-1 (30). Upon cleavage by caspase-1, the N-terminal fragment of GSDMD is liberated and subsequently oligomerizes to form pores in the plasma membrane (31). These pores serve as conduits for the release of mature IL-1β into the extracellular milieu (32). Once outside the cell, IL-1β can bind to its cognate receptors on neighboring cells, initiating and amplifying pro-inflammatory signaling cascades that contribute to the pathogenesis of tendinopathy and other inflammatory conditions (33).

## 3 IL-1β signal transduction

**Figure 3:**
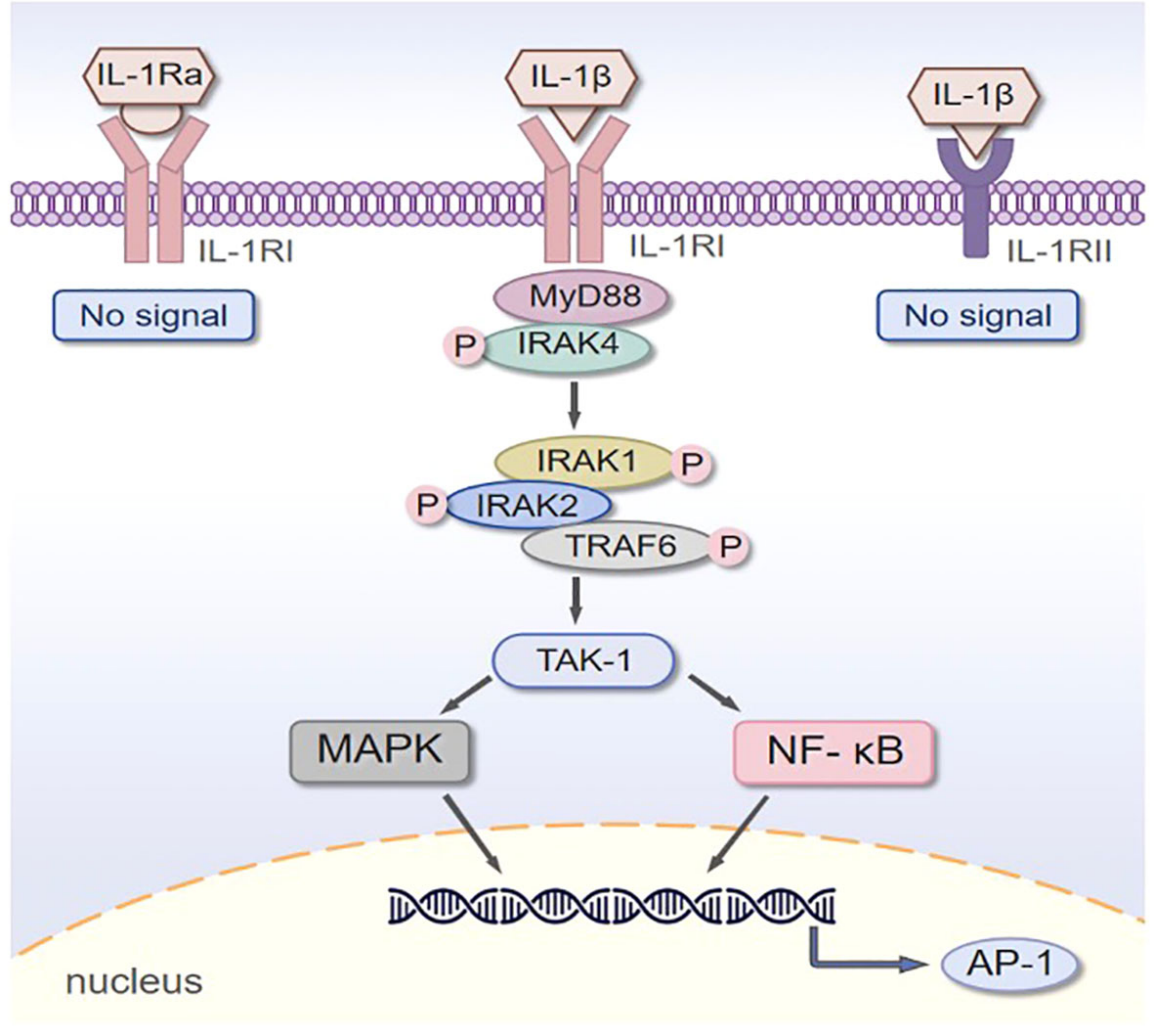
Schematic presentation of IL-1β signal transduction. Left: The IL-1 receptor antagonist (IL-1Ra) binds to the IL-1 type I receptor (IL-1RI), which fails to initiate signal transduction; Middle: IL-1β binding to IL-1RI triggers a functional signaling pathway. This recruits myeloid differentiation primary response protein 88 (MyD88), followed by phosphorylation (“P”) and activation of interleukin-1 receptor-associated kinase 4 (IRAK4). Subsequent phosphorylation of IRAK1, IRAK2, and tumor necrosis factor receptor-associated factor 6 (TRAF6) occurs, with activated TRAF6 inducing the activation of transforming growth factor-β-activated kinase 1 (TAK-1). TAK-1 then activates downstream cascades, including the mitogen-activated protein kinase (MAPK) pathway and the nuclear factor kappa-light-chain-enhancer of activated B cells (NF-κB) pathway. These pathways translocate to the nucleus to regulate gene transcription, such as the induction of activator protein 1 (AP-1); Right: IL-1β binding to the IL-1 type II receptor (IL-1RII) does not transduce a signal.

Upon release, IL-1β activity is tightly regulated through its receptors. IL-1 receptor type I (IL-1RI), expressed ubiquitously on nucleated cells, is essential for initiating signal transduction (34). IL-1β also binds to a second receptor, IL-1 receptor type II (IL-1RII), which functions as a decoy receptor by competing with IL-1RI for ligand binding, thereby negatively regulating IL-1βsignaling. Furthermore, the endogenous IL-1 receptor antagonist (IL-1Ra) binds to IL-1RI with high affinity, preventing downstream signaling activation (35). Ligand binding to IL-1RI initiates the formation of a binary IL-1β:IL-1RI complex. This complex subsequently recruits the interleukin-1 receptor accessory protein (IL-1RAcP), forming the ternary signaling complex IL-1β:IL-1RI: IL-1RAcP (36). The Toll/interleukin-1 receptor (TIR) domains within this complex then engage the TIR domain of the adaptor protein myeloid differentiation primary response 88 (MyD88). MyD88 recruits interleukin-1 receptor-associated kinases (IRAKs), specifically IRAK4, IRAK1, and IRAK2. IRAK4 autophosphorylates and subsequently phosphorylates IRAK1 and IRAK2, enabling their association with tumor necrosis factor receptor-associated factor 6 (TRAF6) (37). TRAF6 serves as an E3 ubiquitin ligase that recruits and activates TGF-β-activated kinase 1 (TAK1). Activated TAK1 phosphorylates components of the IκB kinase (IKK) complex and mitogen-activated protein kinase (MAPK) cascades (38). This leads to the activation of key transcription factors, including: Nuclear factor kappa B (NF-κB), Activator protein-1 (AP-1), and Members of the MAPK family: p38 MAPK, c-Jun N-terminal kinase (JNK), and extracellular signal-regulated kinase (ERK) (39). The activation of these transcription factors induces the expression of target genes involved in diverse cellular responses, ultimately contributing to the pathogenesis and progression of various diseases in a cell-type-specific manner (**Figure 3**).

## 4 Main roles of IL-1β in tendinopathy

**Figure 4:**
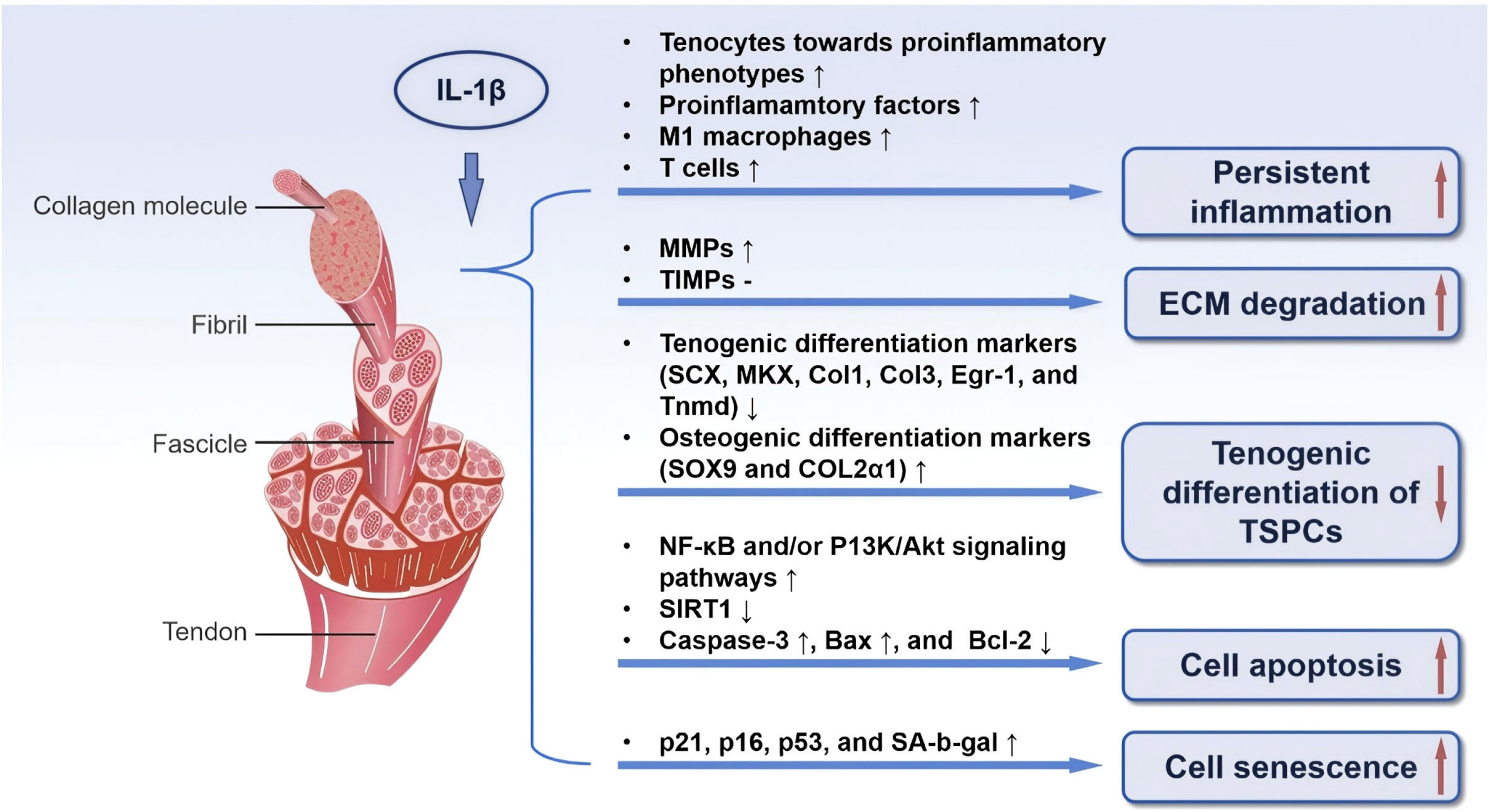
The roles and mechanisms of IL-1β in the progression of tendinopathy. Exposure to IL-1β disrupts tendon homeostasis by triggering a pro-inflammatory microenvironment and key pathological processes. This leads to an imbalance in extracellular matrix (ECM) remodeling, a shift in tendon stem/progenitor cell (TSPC) differentiation away from tenogenesis, and increased cellular apoptosis and senescence. Collectively, these mechanisms drive the progression of tendinopathy. (↑: increase; ↓: decrease).

The pathophysiology of tendinopathy remains incompletely understood. Proposed pathogenic mechanisms include aberrant mechanical loading, sustained inflammatory responses, imbalance between matrix metalloproteinases (MMPs) and their tissue inhibitors (TIMPs), aberrant differentiation of tendon stem/progenitor cells (TSPCs), dysregulated apoptosis and cellular senescence, as well as disruption of collagen fiber architecture characterized by diminished type I collagen synthesis and disorganized deposition of type III collagen (40, 41). Accumulating evidence implicates IL-1β as a significant mediator in these pathological processes (**Figure 4**), as elaborated below.

### 4.1 IL-1β amplifies the inflammatory responses

Persistent inflammation is now recognized as a fundamental driver of tendinopathy pathogenesis, orchestrated by dynamic crosstalk between three interconnected cellular compartments: the stromal (tenocytes, tendon stem/progenitor cells), immune-sensing (resident macrophages, mast cells), and infiltrating (recruited immune cells) compartments. Rather than a transient response, inflammation in tendinopathy reflects a dysregulated, self-sustaining network that disrupts tendon homeostasis (42). Within this network, the cytokine IL-1β emerges as a critical signaling node, mediating a feed-forward inflammatory cascade that engages all three compartments and propagates chronicity (43).

The inflammatory cascade in tendinopathy is frequently initiated within the immune-sensing compartment, where resident mast cells and macrophages recognize microdamage or other pathological stimuli via pattern recognition receptors. Upon activation, these cells release IL-1β, which subsequently acts on both infiltrating immune cells and local stromal components. A central pathogenic mechanism is the capacity of IL-1β to polarize macrophages toward a pro-inflammatory M1 phenotype. These M1 macrophages further amplify IL-1β production and recruit additional immune cells, establishing a self-sustaining inflammatory loop that contributes to chronic tendon degeneration (44). However, the influence of IL-1β on M2 macrophages—a phenotype generally associated with anti-inflammatory and reparative functions—is less clearly defined. Whether IL-1β suppresses M2 differentiation, alters the functional properties of existing M2 macrophages, or disrupts the balance between M1 and M2 populations represents a significant knowledge gap. Elucidating these mechanisms will be essential for understanding the dysregulated repair processes in tendinopathy. Future studies should aim to clarify the temporal and contextual effects of IL-1β on macrophage. Most critically, IL-1β directly reprograms the stromal compartment, driving tenocytes into an activated, pro-inflammatory state (45–47). This phenotypic shift is not merely a passive response but represents a fundamental change in tenocyte identity and function. Evidence from human studies indicates that tenocytes from tendinopathic tissues exhibit a sustained activated phenotype, characterized by elevated expression of markers like podoplanin (PDPN) (45). Notably, this inflammatory memory can persist long-term, as tenocytes isolated from patients’ years after clinical intervention display heightened sensitivity to IL-1β challenge, secreting elevated levels of IL-6 and IL-8. This suggests that IL-1β exposure can epigenetically imprint a hyper-responsive state on tenocytes, a potential mechanism for disease chronicity and recurrence (45). At the molecular level, the sustained inflammatory phenotype is underpinned by constitutive activation of the NF-κB pathway (48). IL-1β signaling robustly activates NF-κB, which not only drives the immediate expression of classic inflammatory mediators (IL-6, IL-8, COX-2, PGE-2) but also appears to lower the threshold for subsequent activation, thereby sensitizing tenocytes (49). Inhibition of NF-κB potently reduces IL-1β-induced cytokine production, confirming its central role (50). Furthermore, IL-1β orchestrates a multi-faceted attack on tendon homeostasis by upregulating prostaglandin E synthase (mPGES) and specifically enhancing the expression of the EP4 receptor for PGE2, creating an autocrine loop that may amplify inflammatory and catabolic signals (50–52). The cytokine also stimulates the release of nociceptive mediators like Substance P, directly linking the inflammatory process to pain (53). The cycle of chronicity is further reinforced by IL-1β’s impact on immune cell recruitment (54). By inducing tenocytes to produce chemokines such as CCL20 and CCL5, IL-1β facilitates the recruitment and activation of T cells. These activated T cells reciprocally produce TNF-α and IL-1β, which further activate tenocytes, establishing a vicious paracrine loop that is difficult to resolve (55). This T cell-tenocyte crosstalk not only perpetuates inflammation but also directly contributes to pathological matrix remodeling, as evidenced by an increased collagen III/I ratio (56).

**Figure 5:**
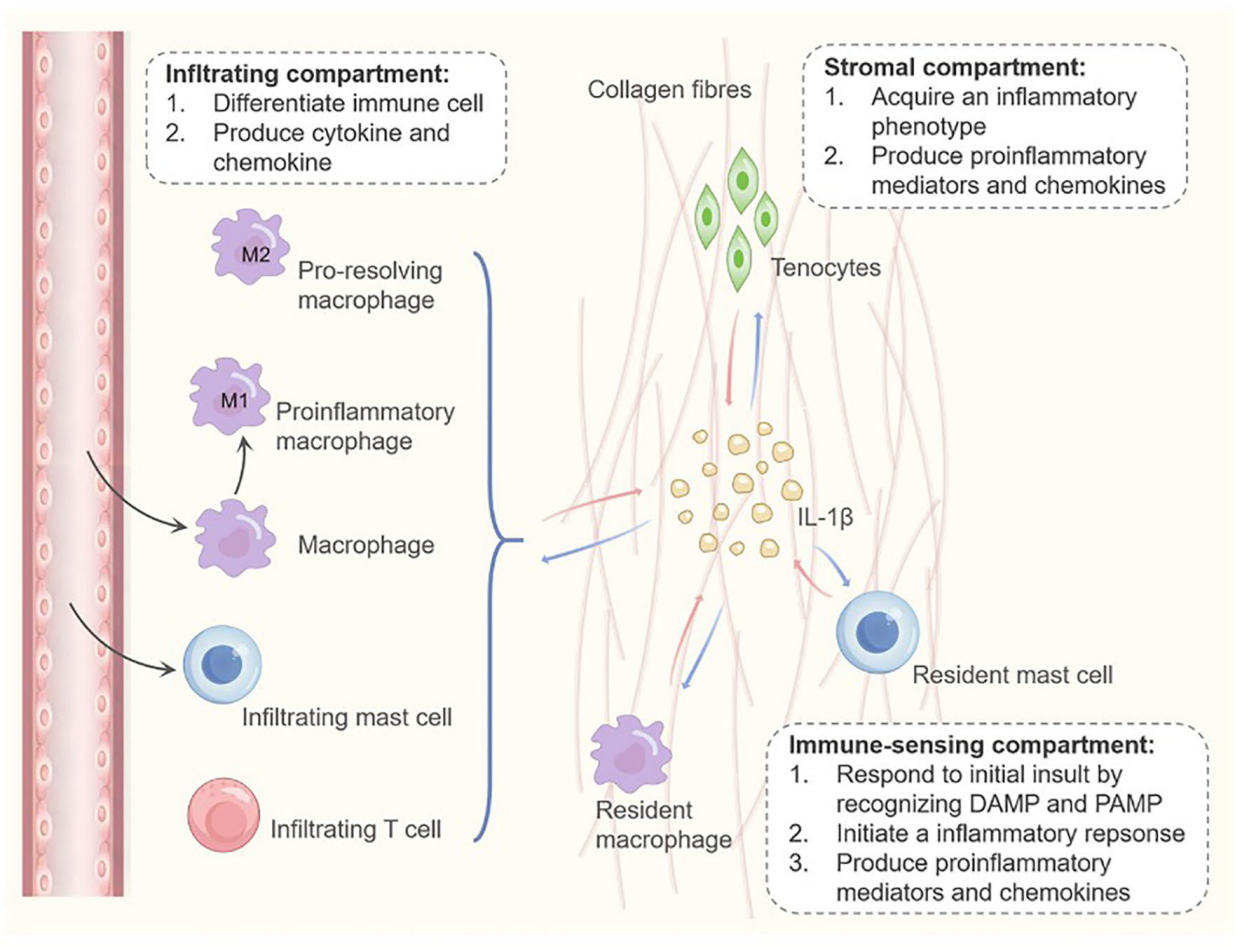
The role of IL-1β in chronic inflammation during degenerative tendinopathy. In tendinopathy, IL-1β—whether derived from stromal tenocytes or immune cells of the immune-sensing or infiltrating compartments—sustains chronic inflammation by promoting macrophage polarization toward an M1 phenotype. This process, in turn, drives stromal tenocytes toward a proinflammatory phenotype, enhances their capacity to secrete proinflammatory mediators, and stimulates chemokine production, thereby facilitating T cell recruitment and activation.

Collectively, IL-1β is a critical node connecting the three cellular compartments and driving chronic inflammation in tendinopathy. It sustains M1 macrophage polarization through a positive feedback loop, induces and maintains the activated inflammatory phenotype of tenocytes, and promotes T cell-mediated immune responses. Future therapeutic strategies could focus on targeting this cytokine network, particularly disrupting the chronic inflammatory state maintained by the IL-1β/NF-κB axis, potentially offering new avenues for the fundamental treatment of tendinopathy (**Figure 5**).

### 4.2 IL-1β induces ECM degradation

The progressive degradation of the extracellular matrix (ECM) is a hallmark of degenerative tendinopathy, with matrix metalloproteinases (MMPs) serving as central executors of ECM catabolism (57). A substantial body of evidence indicates that the pro-inflammatory cytokine IL-1β acts as a primary upstream regulator of MMP expression in tenocytes, thereby disrupting ECM homeostasis. Rather than merely cataloging individual findings, this section synthesizes the mechanistic pathways through which IL-1β drives pathological matrix remodeling and examines its critical interplay with mechanical stimuli—a relationship that underpins a self-perpetuating cycle of tendon degeneration.

IL-1β exerts its catabolic effects by shifting the delicate balance between MMPs and their endogenous inhibitors, the tissue inhibitors of metalloproteinases (TIMPs) (58). In healthy tendons, TIMP levels predominate, ensuring controlled turnover. However, upon IL-1β stimulation, tenocytes from multiple species exhibit a coordinated upregulation of key collagenases and stromelysins—notably MMP-1, MMP-3, and MMP-13—without a commensurate increase in TIMP-1 or TIMP-2 (59, 60). This selective induction creates a proteolytic imbalance favoring net matrix degradation. Mechanistically, IL-1β signaling converges on several key pathways: binding to the IL-1 receptor activates the JNK/SAPK and broader MAPK cascades (ERK1/2, p38, JNK), which are requisite for maximal MMP transcription (61, 62). The pathological relevance of this axis is further underscored by the ability of IL-1Ra to attenuate collagen breakdown, albeit at a higher concentration due to competitive receptor binding (63). A particularly insightful concept emerging from the literature is the positive feedback loop initiated by IL-1β. Beyond directly inducing MMPs, IL-1β can stimulate tenocytes to produce more endogenous IL-1β, thereby amplifying and prolonging the catabolic signal (9). This autocrine/paracrine loop may explain the progressive nature of tendinopathy even after the initial insult has subsided. Furthermore, the relative potency of IL-1β compared to other cytokines like TNF-α suggests a specialized role: while TNF-α may initiate degradation, IL-1β appears more critical for driving its progression, as evidenced by its superior capacity to upregulate MMP-3 and MMP-13 in ex vivo models (64).

Perhaps the most significant advance in understanding IL-1β’s role in tendinopathy lies in its integration with the condition’s biomechanical etiology (65). Mechanical overload, a primary risk factor, is no longer viewed as merely causing structural fatigue, but as a trigger for a complex biologic response (66). In this context, tenocytes function as key mechanosensory units that transduce mechanical stimuli into molecular signals, modulating ECM synthesis and degradation to establish novel homeostatic setpoints adapted to their mechanical environment (67). This mechanoresponsive capacity is governed by the cytoskeletal architecture and its dynamic interactions with the ECM (68), where α-smooth muscle actin (αSMA)-mediated organization generates intrinsic cellular tension (69). This tension propagates to the ECM as contractile traction forces, establishing a biomechanical equilibrium within the tissue microenvironment (70). However, pathological overload disrupts this equilibrium: microdamage from overloading relieves mechanical tension on tenocytes, effectively creating a state of “unloading” at the cellular level (71). This aberrant mechanical environment not only stimulates IL-1β production—potentially via mechanisms such as H_2_O_2_-induced NLRP3 inflammasome activation (72, 73)—but also critically determines cellular responsiveness to the cytokine. It has been reported that tenocytes with diminished intrinsic tension exhibit exaggerated catabolic responses to IL-1β, including markedly increased MMP expression and apoptosis, ultimately leading to ECM destruction (74). Conversely, tenocytes maintaining high cellular tension demonstrate markedly attenuated responses to IL-1β-induced catabolic events (75), establishing cellular tension state as a pivotal regulator of IL-1β-driven pathology.

Emerging evidence further suggests that dysregulation of mechanosensitive signaling pathways, such as the Yes-associated protein (YAP) pathway, may link abnormal mechanical loading to inflammatory and catabolic responses in tenocytes. Under physiological loading, YAP shuttles between the cytoplasm and nucleus to regulate cell proliferation and matrix homeostasis; however, excessive mechanical stress can lead to aberrant YAP activation, which has been associated with pro-inflammatory gene expression and matrix remodeling (76). Although direct crosstalk between YAP and IL-1β in tendinopathy remains underexplored, studies in other musculoskeletal tissues suggest that YAP signaling can modulate IL-1β production and subsequent MMP activation (77). The resulting cytokine surge promotes MMP secretion, degrading the pericellular matrix and further compromising tissue integrity. Thereby, a self-perpetuating vicious cycle is established: mechanical damage induces IL-1β expression and reduces cellular tension, which synergistically promotes ECM breakdown; this degradation, in turn, weakens the tendon structure and increases its susceptibility to additional mechanical injury. Notably, both excessive strain and stress-shielding upregulate IL-1β and MMPs, thereby driving degenerative processes. The magnitude of mechanical stretch plays a decisive role in determining cellular responses: while low-magnitude strain may exert protective or anti-inflammatory effects, high-magnitude strain acts synergistically with IL-1β to markedly upregulate MMP-1 and MMP-3 expression (78). This synergy underscores that mechanical stress and inflammatory signaling are integrated at the molecular level, collectively governing the net catabolic outcome in tendon tissue. Importantly, pharmacological restoration of cytoskeletal tension (e.g., via calyculin A administration (79)) has been shown to reverse IL-1β-driven catabolism by reestablishing physiological tension homeostasis, highlighting targeted modulation of tenocyte mechanobiology as a promising therapeutic strategy for counteracting inflammatory degradation in tendinopathy.

Collectively, these studies firmly establish IL-1β as a master regulator of ECM degradation in tendinopathy, primarily by disrupting the critical balance between MMPs and TIMPs. The interplay between IL-1β and mechanical loading is particularly noteworthy, suggesting a vicious cycle where microdamage from overloading induces IL-1β expression, which in turn promotes further ECM cleavage, weakening the tendon and making it more susceptible to additional damage. Critically, the cellular response to IL-1β is governed by the mechanobiological state of tenocytes: loss of intrinsic cytoskeletal tension following microdamage hypersensitizes cells to IL-1β, exacerbating matrix destruction. Future therapeutic strategies should extend beyond anti-inflammation to target this mechanobiological dysregulation. Elucidating the crosstalk between pathways like YAP and IL-1β signaling, and developing interventions to “re-tension” tenocytes, hold promise for breaking the degenerative cycle and promoting tendon regeneration.

### 4.3 IL-1β inhibits tenogenic differentiation of tendon stem/progenitor cell

Beyond the well-established role of tenocytes in tendon homeostasis, the identification of tendon stem/progenitor cells (TSPCs) has fundamentally expanded our understanding of tendon biology and pathology (80). TSPCs, distinct from mature tenocytes, possess the critical capacities for self-renewal and differentiation, serving as a reservoir for tissue maintenance and repair (81). In healthy tendon healing, TSPCs undergo tenogenic differentiation to regenerate functional tissue (82). However, in the pathological microenvironment of tendinopathy, TSPCs are driven toward aberrant differentiation lineages—namely chondrogenic, osteogenic, and adipogenic—leading to the characteristic degenerative features of lipid deposition, proteoglycan accumulation, and ectopic calcification (83). A key insight emerging from recent research is that the cytokine IL-1β acts as a potent pathological switch that redirects TSPC fate away from regeneration and toward degeneration (84).

It has been reported that IL-1β consistently suppresses the expression of key tenogenic markers such as scleraxis (Scx), tenomodulin (Tnmd), and type I collagen, while simultaneously promoting markers of non-tenogenic lineages. What makes this effect particularly consequential for chronic tendinopathy is its potential persistence. Studies indicate that even transient exposure to IL-1β can cause a lasting impairment of TSPCs’ tenogenic potential, suggesting that an early inflammatory insult may irreversibly compromise the tendon’s intrinsic repair capacity long after the initial cytokine signal has subsided (85). This provides a plausible cellular mechanism for the progressive and often irreversible nature of advanced tendinopathy. The mechanistic underpinnings of this fate switch involve multiple signaling pathways. IL-1β has been shown to act through the downregulation of microRNAs such as miR-337-3p, leading to the activation of Nox4-JNK and IRS1-ERK cascades that favor chondro-osteogenic differentiation (86). Other work implicates the Rac1 signaling pathway in IL-1β-mediated induction of osteogenic markers (SOX9, COL2α1) and suppression of tenogenic factors (87). Furthermore, the source of IL-1β is also of pathological relevance. Recent evidence links pyroptotic macrophages—a highly inflammatory form of cell death—to IL-1β release, which in turn drives TSPCs toward osteogenic differentiation, creating a direct bridge between immune cell activity and degenerative tissue remodeling (88).

In summary, the impact of IL-1β on TSPCs represents a critical paradigm shift in tendinopathy pathogenesis. It moves the focus beyond the catabolic degradation of the existing matrix by tenocytes to include the failure of regenerative potential through the maldifferentiation of the progenitor cell pool. This dual attack—simultaneously breaking down mature tissue and corrupting the cells meant to rebuild it—explains the progressive and structurally disastrous nature of the disease. Therefore, therapeutic strategies that not only block IL-1β’s catabolic effects but also safeguard or restore the tenogenic differentiation capacity of TSPCs could be pivotal in achieving true tendon regeneration rather than mere symptom mitigation. Future research should prioritize elucidating the epigenetic mechanisms behind IL-1β’s long-lasting effects on TSPCs and identifying strategies to therapeutically reverse this pathological reprogramming.

### 4.4 IL-1β accelerates cell apoptosis

Beyond its well-characterized roles in promoting inflammation and matrix degradation, IL-1β contributes to tendinopathy pathogenesis by accelerating tenocyte death through the induction of apoptotic pathways (89). While physiological apoptosis is essential for tissue turnover, its dysregulation represents a critical mechanism of cellular depletion and functional decline in degenerative tendons (90). A growing body of evidence positions IL-1β as a master regulator of this pathological apoptosis, primarily through its orchestration of the NF-κB and PI3K/Akt signaling axes.

The pro-apoptotic effect of IL-1β is executed via the classic mitochondrial pathway, culminating in the activation of caspase-3, the key effector protease of apoptosis. IL-1β signaling shifts the delicate balance between pro-apoptotic and anti-apoptotic proteins, consistently upregulating Bax while suppressing Bcl-2 (91). This imbalance promotes mitochondrial membrane permeabilization, triggering the caspase cascade and committing the cell to death (91). What elevates this finding from a simple observation to a mechanistically insightful one is the identification of the specific signaling pathways involved and their regulatory nodes. Research demonstrates that IL-1β concurrently activates both the NF-κB and PI3K/Akt pathways to drive apoptosis. Notably, the caspase-3-dependent apoptotic process appears specifically tied to NF-κB activation (92). A crucial upstream regulator of this pathway is Sirtuin 1 (SIRT1), a NAD+-dependent deacetylase associated with cellular stress responses (93). IL-1β downregulates SIRT1, which in turn unleashes NF-κB activity, leading to the pro-apoptotic Bax/Bcl-2 dysregulation (93). This mechanistic hierarchy is substantiated by interventional studies: compounds like chitosan and resveratrol can attenuate IL-1β-induced apoptosis by activating SIRT1, thereby suppressing NF-κB signaling and restoring cell survival (92, 94).

In summary, the induction of apoptosis solidifies IL-1β’s role as a central mediator of tendon degeneration. Its ability to activate specific, interconnected pathways like NF-κB and PI3K/Akt, finely tuned by regulators like SIRT1, provides a sophisticated mechanism for excessive cell death. Viewing IL-1β through the lens of apoptosis unveils new therapeutic opportunities aimed at preserving the tenocyte population by targeting these regulatory nodes, potentially slowing disease progression by maintaining the cellular workforce essential for tendon homeostasis.

### 4.5 IL-1β stimulates cell senescence

Of note, cellular senescence has emerged as a pivotal mechanism in tendinopathy, operating in concert with apoptosis to drive tissue dysfunction (95). Senescent cells, characterized by irreversible growth arrest and a distinct secretory phenotype, accumulate in degenerative tendons and contribute to an impaired regenerative environment (96). Key biomarkers of this state include elevated expression of cyclin-dependent kinase inhibitors p16 and p21, tumor suppressor p53, and increased senescence-associated β-galactosidase (SA-β-gal) activity (97). A compelling body of evidence now identifies IL-1β as a potent inducer of this deleterious state in both tenocytes and tendon stem/progenitor cells (TSPCs).

The mechanistic link between IL-1β and senescence is robustly demonstrated across multiple studies. Stimulation with IL-1β reliably triggers a canonical senescent phenotype in tenocytes, manifesting as altered morphology, growth arrest, and upregulated expression of p16, p21, and p53, alongside enhanced SA-β-gal activity (98). A key insight is the central role of the NF-κB signaling pathway in mediating this response. The pathological relevance of this axis is confirmed by interventional data: inhibition of NF-κB signaling effectively attenuates IL-1β-induced senescence markers in TSPCs. Furthermore, the microRNA miR-146a, a known negative regulator of inflammation, has been shown to confer protection against IL-1β-driven senescence by suppressing the upstream IRAK4/TRAF6/NF-κB cascade (99). This not only reinforces the role of NF-κB but also suggests the existence of endogenous regulatory mechanisms that become overwhelmed in the diseased state.

In summary, IL-1β’s capacity to induce cellular senescence solidifies its position as a master regulator of tendon degeneration, acting through a well-defined NF-κB-dependent pathway. This mechanism contributes to the failure of tissue homeostasis not merely by reducing cell numbers, as in apoptosis, but by creating a population of dysfunctional, senescent cells that actively degrade the tissue milieu. Therapeutic strategies aimed at selectively eliminating senescent cells (senolytics) or modulating their secretory phenotype (senomorphics) may therefore hold promise for disrupting this vicious cycle in IL-1β-driven tendinopathy.

## 5 Conclusions and future perspective

In summary, the collective evidence establishes IL-1β as a pivotal mediator in tendinopathy pathogenesis. The data synthesized herein demonstrate that IL-1β contributes to disease progression through multiple mechanisms: potentiating inflammatory responses, mediating extracellular matrix (ECM) degradation, suppressing tenogenic differentiation of tendon stem/progenitor cells (TSPCs), accelerating cellular apoptosis, and promoting senescence. While the reviewed studies provide valuable insights into IL-1β’s pathogenic mechanisms and identify promising therapeutic targets, significant knowledge gaps persist.

A key challenge in interpreting current research on IL-1β mechanisms in tendinopathy lies in the widespread use of heterogeneous tenocyte populations lacking subtype-specific markers. Although *in vitro* models offer valuable molecular insights that may inform clinical prevention strategies, the physiological relevance of findings can be limited when tenocytes are isolated using conventional methods, typically enzymatic digestion of whole tendon tissue, which captures a mixed population of cells with potentially distinct roles (100). Without markers to distinguish tenocyte subtypes (e.g., resident tendon stem/progenitor cells, mature tenocytes, or fibrotic precursors), it remains difficult to attribute IL-1β responses to specific subpopulations (101). This heterogeneity may obscure important functional differences in inflammatory sensitivity, matrix turnover, and mechanoresponsiveness, thereby complicating the extrapolation of *in vitro* results to *in vivo* pathology. Furthermore, many studies focus predominantly on transcriptional-level alterations induced by IL-1β, while comprehensive analyses of functional protein dynamics, including post-translational modifications, protein activity, and turnover, are still limited. This is particularly relevant in the context of IL-1β-driven tendinopathy, as IL-1β not only regulates gene expression but also influences the activation and stability of key effector proteins such as matrix MMPs and inflammatory mediators through post-translational mechanisms. For example, MMP activity is often controlled by proteolytic activation and inhibition, processes that are not fully captured by mRNA measurements (102). A greater emphasis on protein-level dynamics will be essential to fully understand the functional impact of IL-1β signaling on tendon degradation and repair. Collectively, future studies should prioritize the identification of tenocyte subtype-specific markers through single-cell transcriptomic or proteomic approaches, enabling more precise cell sorting and functional characterization. In parallel, integrating multi-omics strategies that assess not only transcriptional changes but also protein expression, modification, and metabolic activity will provide a more holistic view of IL-1β’s role in tendinopathy.

Secondly, chronic inflammation in tendinopathy demonstrates intricate interplay with other proinflammatory cytokines, notably TNF-α and IL-6 (103, 104). Consequently, therapeutic strategies targeting a single cytokine may prove inadequate for complete disease resolution, potentially explaining limited clinical efficacy. This premise is substantiated by recent findings wherein concomitant exposure of equine tenocytes to IFN-γ, TNF-α and IL-1β synergistically amplified MMP-1, MMP-3, and MMP-13 expression—an effect unmitigated by IL-1Ra administration (15). This indicates IL-1β inhibition alone is insufficient to counterbalance the catabolic influence of other inflammatory mediators. Furthermore, independent inhibition of TNF-α or IL-6 signaling has demonstrated therapeutic potential in tendinopathy models (105, 106). Given this collective evidence, synergistic inhibition of IL-1β with other pathogenic cytokines represents a promising therapeutic approach warranting systematic investigation.

Finally, while the primary focus of this review has been on the mechanistic role of IL-1β in tendinopathy, the translational prospects of targeting this cytokine warrant discussion. Currently, there are no IL-1β-targeted therapies specifically approved for the treatment of tendinopathy. However, potent biologic agents that neutralize IL-1β, such as the monoclonal antibody Canakinumab, or block its receptor, such as Anakinra (a recombinant IL-1 receptor antagonist), are clinically available and successfully used for other inflammatory conditions, including rheumatoid arthritis and gouty arthritis (107, 108). This established safety profile presents a compelling rationale for investigating their repurposing for tendinopathy. Despite this potential, their application to tendinopathy faces challenges. The translation of IL-1β blockade to tendinopathy is not straightforward, as the pathology often involves a complex interplay of inflammatory, degenerative, and failed healing processes. A key question is identifying the patient subgroup most likely to benefit from anti-IL-1β therapy, presumably those in the earlier, more inflammatory stages of the disease. Furthermore, optimal delivery methods—systemic versus localized, single injection versus sustained-release formulations—need careful evaluation to maximize efficacy and minimize systemic side effects.
