# Correction: Mechanistic insights into IL-1β-mediated progression of tendinopathy

**DOI:** 10.3389/fimmu.2025.1736822

**Published:** 2025-11-18

**Authors:** Yuchi Zhang, Jiayue Wang, Fanbo Tang, Runzhi Xian, Huanhuan Zhang, Yanlin Yuan, Guiquan Chen, Guoqiang Yang

**Affiliations:** 1 School of Physical Education, Southwest Medical University, Luzhou, China; 2 Acupuncture and Rehabilitation Department, The Affiliated Traditional Chinese Medicine Hospital of Southwest Medical University, Luzhou, China

**Keywords:** IL-1β, tendinopathy, pathogenesis, therapeutic targets, proinflammatory cytokines

The order of authors in the author list of the published paper was erroneously given as:

“Yuchi Zhang, Jiayue Wang, Fanbo Tang, Runzhi Xian, Huanhuan Zhang, Yanlin Yuan, Guoqiang Yang and Guiquan Chen”

The correct author list reads:

“Yuchi Zhang, Jiayue Wang, Fanbo Tang, Runzhi Xian, Huanhuan Zhang, Yanlin Yuan, Guiquan Chen and Guoqiang Yang”

The original version of this article has been updated.

